# Astaxanthin Induces Transcriptomic Responses Associated with Lifespan Extension in *Caenorhabditis elegans*

**DOI:** 10.3390/antiox11112115

**Published:** 2022-10-27

**Authors:** Feng Ding, Yan Zhao

**Affiliations:** 1 School of Chemistry and Chemical Engineering, Harbin Institute of Technology, Harbin 150001, China; 2 Department of Bioengineering, Harbin Institute of Technology, Weihai 264209, China

**Keywords:** astaxanthin, *C. elegans*, transcriptome, microRNA, lifespan

## Abstract

Astaxanthin is a marine xanthophyll carotenoid which effectively prevents intracellular oxidative stress and has beneficial effects against various human diseases. It has been shown that astaxanthin protects *Caenorhabditis elegans* (*C. elegans*) from oxidative damages and extends the lifespan of *C. elegans* possibly by modulating genes involved in insulin/insulin-like growth factor (IGF) signaling (IIS) and the oxidoreductase system, although the exact mechanisms remain elusive. In this study, RNA sequencing analyses were employed to identify the differentially expressed genes in *C. elegans* in response to astaxanthin treatment. A total of 190 mRNAs and 6 microRNAs (miRNAs) were significantly changed by astaxanthin treatment in *C. elegans*. Gene ontology (GO) term and Kyoto Encyclopedia of Genes and Genomes (KEGG) pathway analyses indicated that the mRNAs and miRNAs significantly altered by astaxanthin mainly function in innate immunity, lipid metabolism and stress responses, a significant portion of which are related to lifespan regulation in *C. elegans*. The study revealed novel mRNA and miRNA targets of astaxanthin, providing new insights for understanding the anti-aging mechanisms and the biological function of astaxanthin.

## 1. Introduction

Astaxanthin is a marine xanthophyll carotenoid with a strong antioxidant property, which is widely applied in the nutraceutical, cosmetics, food and feed industries [1,2,3]. Studies have shown that astaxanthin has beneficial effects against a variety of disease conditions, such as cancer, hypercholesterolemia and diabetes [2], yet the mechanisms remain elusive. It was demonstrated that astaxanthin treatment prolonged the lifespan in a model organism *Caenorhabditis elegans* (*C. elegans*) [4]. The longevity-promoting effects of astaxanthin have been associated with the increase in antioxidant capacity and modulation of key genes in the insulin/insulin-like growth factor (IGF) signaling (IIS) pathway, including *daf-16* and *daf-2* [4,5]. It has also been shown that astaxanthin treatment represses the target of the rapamycin (TOR) pathway through the down-regulation of *let-363* and induces autophagy through the up-regulation of transcription factor EB (TFEB) orthologue *hlh-30* [6,7]. However, it is unclear how astaxanthin regulates the expression of these longevity genes, and while most of the studies have focused on the alteration of messenger RNA (mRNA), few have assessed the changes in non-coding RNAs (ncRNAs). Thus, to better understand the anti-aging mechanisms of astaxanthin, global transcriptomic changes in response to astaxanthin need further assessment.

NcRNAs comprise a variety of RNA species, including microRNAs (miRNAs), tRNA-derived small RNAs (tsRNAs), ribosomal RNAs (rRNAs), PIWI-interacting RNAs (piRNAs), circular RNAs (circRNAs) and long ncRNAs (lncRNAs), which regulate many aspects of cellular processes [8], including cellular proliferation, apoptosis, differentiation and metabolism [9]. Among the small ncRNAs, miRNAs bind to target mRNA with partial base-pair complementarity, resulting in the post-transcriptional repression [10] or activation [11] of target gene expression and affecting a wide range of cellular functions [12]. The total amount of miRNAs declines during aging and the expression of many miRNAs displays age-dependent changes [8]. The heterochronic miRNA *lin-4* is the first ncRNA reported to influence longevity in *C. elegans*, loss of which leads to the reduction of lifespan [13]. Similarly, the loss-of-function mutant of miR-71, miR-238 or miR-246 is short-lived, while the mutation of miR-239 leads to a longer lifespan. Evidence suggests that miR-71 and miR-239 affect longevity through the modulation of genes in the IIS pathway [14]. It is currently unknown whether the anti-aging effects of astaxanthin are associated with alterations in miRNA expression. 

RNA-Sequencing (RNA-seq) is a powerful technique for analyzing transcriptome-wide differential gene expression as well as examining the fine structures of transcriptome, including the detection of novel transcripts, allele-specific expression and splice junctions [15]. To further explore how astaxanthin promotes longevity, we performed RNA-seq on *C. elegans* treated with astaxanthin and analyzed the transcriptomic changes in mRNA and miRNA. Through the identification of the differentially expressed genes (DEGs) and by carrying out gene ontology (GO) and Kyoto Encyclopedia of Genes and Genomes (KEGG) pathway analyses, the study revealed novel genes and pathways targeted by astaxanthin, providing insight into the mechanisms underlying the lifespan extension of astaxanthin. 

## 2. Materials and Methods

### 2.1. Materials

Astaxanthin was purchased from Sigma Chemical (St. Louis, MO, USA). DMSO was purchased from the Beyotime Institute of Biotechnology (Shanghai, China). TRIzol Reagent and SuperScript^®^ II reverse transcriptase were obtained from Invitrogen (Eugene, OR, USA). The quantitative real-time PCR (qRT-PCR) system was obtained from Takara Bio Inc. (Shiga, Japan).

### 2.2. Nematode Strains 

Wide type *C. elegans* N2, obtained from the Caenorhabditis Genetic Centre (CGC), were cultured at 16 °C on solid NGM with *E. coli* OP50 as a food source. To achieve synchronization, worms in their oviposition stage were transferred to NGM plates without OP50 to lay eggs overnight and were removed afterwards. L1-stage worms hatched from the eggs were then transferred to new NGM plates until they reached the adult stage.

### 2.3. Lifespan Assay

Age-synchronized worms at the adult stage were transferred to NGM plates containing 0, 5, 10, 20, 40 and 80 μg/mL of astaxanthin in OP50. Astaxanthin was dissolved in DMSO and the final concentration of DMSO was 0.1%. Approximately 75 nematodes were used for each treatment group. Live worms were transferred to fresh NGM plates every 2 days and counted every day until all of the worms were dead. Worms were scored as dead when they did not respond to repeated touching with a platinum wire. Three independent experiments were performed.

### 2.4. Sample Preparation

Age-synchronized worms at the adult stage were transferred to NGM plates with or without astaxanthin. Worms were transferred to fresh NGM plates every 2 days and were harvested after treatment with astaxanthin for 6 days. The worms were collected and washed with M9 buffer 3 times. Three independent replicates, each with approximately 200 worms, were used in the experiment. Total RNA was extracted with TRIzol Reagent according to the manufacturer’s protocol and stored at −80 °C.

### 2.5. RNA-seq

RNA was quantified using NanoDrop ND-1000 (NanoDrop, Wilmington, DE, USA) and the integrity of RNA was assessed by Agilent 2100. Samples with an RIN number >7.0 were used in the following experiments. Ribosomal RNA was depleted using a Ribo-Zero™ rRNA Removal Kit (Illumina, San Diego, CA, USA). The cleaved RNA fragments were reverse-transcribed and the cDNAs were used to synthesize U-labeled second-stranded DNAs. The ligated products were amplified with PCR. The average insert size for the final cDNA library was 300 bp (±50 bp). The paired-end sequencing was performed on an Illumina Hiseq 4000 (LC Bio, Hangzhou, China) following the protocol recommended by the vendor and the DEGs were analyzed using R package (version 3.2.5) edgeR.

### 2.6. Analyses of RNA-seq Datasets

Transcript quantification was calculated with FPKM (expected number of Fragments Per Kilobase of transcript sequence per Million base pairs sequenced) and FPKM value of 1 was defined as the threshold for transcript expression [16]. Volcano plot, heatmap, the enrichment of GO terms and KEGG pathways and Gene Set Enrichment Analyses (GSEA) were carried out using omicstudio (https://www.omicstudio.cn/index, accessed on 14 July 2022). A total of 394 genes (*p* < 0.1) altered by astaxanthin treatment were used for GO terms and KEGG analyses. The gene set files used for GSEA were downloaded from Gong et al. [17].

### 2.7. PPI network Construction

Protein–protein interaction (PPI) was employed to analyze the interrelationship among 190 significant genes (*p* < 0.05). The Search Tool for the Retrieval of Interacting Genes (STRING) database (https://www.string-db.org/, accessed on 16 July 2022) was used to predict the interrelationships of proteins. The PPI network was visualized by Cytoscape software.

### 2.8. miRNA Target Prediction

In order to find possible miRNA targets, RNA target genes were predicted based on the full length of mRNA sets using Targetscan software. 

### 2.9. Oil Red O Staining

Worms treated with or without astaxanthin for 6 days were collected and washed three times with M9 buffer. After fixation with 4% paraformaldehyde, worms were resuspended and dehydrated in 60% isopropanol. The samples were then incubated with Oil Red O staining solution overnight at room temperature [18]. After staining, the worms were examined using an Optec BDS200 inverted microscope. Image-Pro Plus was used to quantify the Oil Red O staining. At least 25 worms were counted for each group. Three independent experiments were performed.

### 2.10. Quantitative RT-PCR Analyses

Three independent replicates, each with approximately 200 worms, were used to prepare RNA samples. Total RNA was extracted using Trizol reagent and cDNA was synthesized using reverse transcriptase (SuperScript^®^ II). QRT-PCR was performed using TB Green^®^ Premix Ex Taq™ II (Tli RNaseH Plus) and ABI 7500/7500-Fast Real-Time PCR System according to the manufacturer’s protocol. The expression of *pmp-3* was used as the internal control. The primers used in qRT-PCR were listed in Table S1.

### 2.11. Statistical Analysis

The data of the lifespan experiments were analyzed using the Kaplan–Meir survival analysis of SPSS 22.0. The statistical analyses of other data were performed by one-way ANOVA unless mentioned otherwise. The *p* values less than 0.05 were considered as statistically significant.

## 3. Results

### 3.1. Astaxanthin Extends the Lifespan of C. elegans

**Figure 1:**
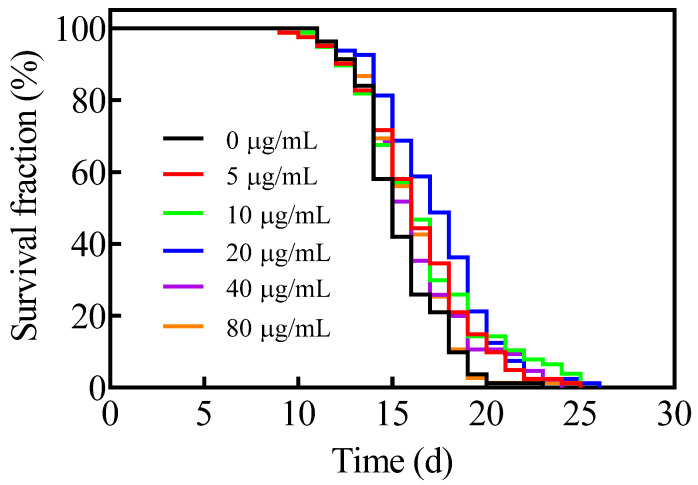
Effects of astaxanthin on the lifespan of *C. elegans*. Worms synchronized to adult stage were treated with different concentrations of astaxanthin (0, 5, 10, 20, 40, 80 µg/mL).

First, the effects of astaxanthin (0, 5, 10, 20, 40, 80 µg/mL) on the lifespan of *C. elegans* were examined. As shown in Figure 1 and Table 1, the mean lifespans of the worms treated with 5–20 µg/mL of astaxanthin were significantly increased in comparison with that of the control group. The mean and maximum lifespans of worms treated with 20 µg/mL of astaxanthin were extended 12.3% and 12.0%, respectively. The higher doses of astaxanthin (40 and 80 µg/mL) appeared less effective for extending the lifespan. Thus, 20 µg/mL was chosen for the treatment of astaxanthin in subsequent experiments.

### 3.2. Differentially Expressed Genes Affected by Astaxanthin in C. elegans

**Figure 2:**
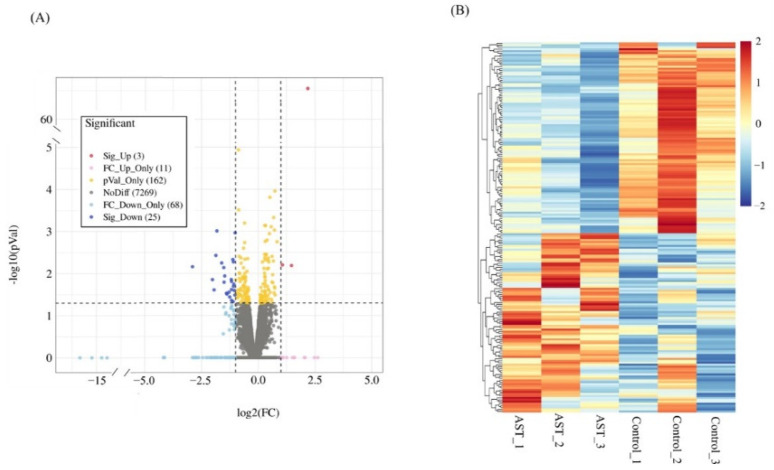
Volcano plot and heatmap of genes altered by astaxanthin treatment in *C. elegans*. (**A**) Volcano plot. Each point represents a single gene. Plotted along the x-axis is the log2(FC) of each gene (comparison of gene expression in astaxanthin-treated worms with the untreated worms). The y-axis represents the negative logarithm of the corresponding *p*-value of that gene. Up- and down-regulated (3 and 25, respectively) genes with *p* < 0.05 and |log2FC| ≥ 1 are shown in red and blue, respectively, whereas genes showing no differential expression are shown in grey. Up- and down-regulated (11 and 68, respectively) genes with |log2FC| ≥ 1 but *p* ≥ 0.05 are shown in light red and light blue, respectively, and 162 genes changed significantly, but with |log2FC| < 1, and are shown in yellow. (**B**) Heatmap of significantly changed genes. Color correlates with the value of z-score. Z-score = (X − mean)/SD. AST, astaxanthin.

To identify the changes in the transcriptional profile of *C. elegans* exposed to astaxanthin, RNA-seq was performed using worms treated with or without astaxanthin (20 μg/mL) for 6 days. Using *p* < 0.05 and fold change |log2FC| ≥ 1 as cut-off criterion, 22 DEGs with statistical significance that can be functionally annotated were shown in Table 2. The overall distribution of altered genes with FPKM > 1 was shown in the volcano plot (Figure 2A). DEGs (*p* < 0.05) were presented in a heatmap (Figure 2B) and shown in Table S2.

### 3.3. Enrichments of GO Terms and KEGG Pathways in the Transcriptomes of Astaxanthin-Treated C. elegans

**Figure 3:**
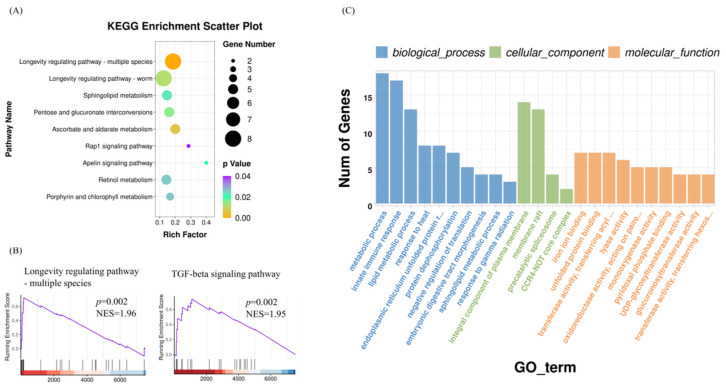
Significant KEGG pathways identified by enrichment analyses (**A**) and GSEA (**B**) (*p* < 0.05, *q* < 0.25) in *C. elegans* treated with astaxanthin. Rich factor is the ratio of the DEG number in each group to the total number of genes identified in the KEGG analyses. Normalized enrichment score (NES) reflects the enrichment of a pathway in the ranked gene list after correction for the size of the pathway. (**C**) Significant GO terms of DEGs found by enrichment analyses in *C. elegans* treated with astaxanthin.

To examine the biological function altered by astaxanthin treatment in *C. elegans*, the gene expression profile from RNA-seq was studied by KEGG and GO term enrichment analyses. A total of nine KEGG pathways were found to be significantly changed by astaxanthin treatment in *C. elegans* (*p* < 0.05) (Table S3). As expected, the longevity regulating pathways (longevity regulating pathway–multiple species (ko04213) and longevity regulating pathway–worm (ko04212)) were among the most significant KEGG pathways enriched (Figure 3A). GSEA analysis considers the whole-genome, and preserves gene–gene correlations, thus providing a more accurate null model [19]. When GSEA analyses were used to analyze the DEGs, the significant KEGG pathways identified in *C. elegans* treated with astaxanthin were longevity regulating pathway-multiple species (ko04213) and TGF-beta signaling pathway (ko04350) (Figure 3B), which plays many important biological roles in *C. elegans*, including the regulation of aging and longevity [20]. GO term enrichment resulted in 49 significant terms changed by astaxanthin treatment (Table S4). Among them, the most highly represented GO terms altered by astaxanthin treatment in *C. elegans* included the metabolic process (GO:0008152), innate immune response (GO:0045087), lipid metabolic process (GO:0006629), heat-shock proteins (enriched in response to heat (GO:0009408) and endoplasmic reticulum unfolded protein response (GO:0030968)), cytochrome P450 family (enriched in iron ion binding (GO:0005506) and oxidoreductase activity (GO:0016705)) and UDP-glucuronosyltransferase activity (UGT) (GO:0008194) (Figure 3C). The alterations of these biological functions have all been shown to affect lifespan [21]. It was noticed that the majority of genes enriched in metabolic process (GO:0008152) were related to lipid and fatty acid metabolism. Both GO and KEGG enrichment analyses identified sphingolipid metabolism (GO:0006665 and ko00600). Similarly, genes with molecular functions related to UGT were enriched in both KEGG pathways (pentose and glucuronate interconversions (ko00040), ascorbate and aldarate metabolism (ko00053) and porphyrin and chlorophyll metabolism (ko00860)) and GO terms (UDP-glucuronosyltransferase activity (UGT) (GO:0008194), glucuronosyltransferase activity (GO:0015020) and transferase activity, transferring hexosyl groups (GO:0016758)). Taken together, astaxanthin may modulate the expression of a series of genes, affecting signal pathways and biological functions associated with aging and longevity, some of which play important roles in lifespan regulation, eventually promoting longevity in astaxanthin-treated *C. elegans*.

### 3.4. PPI Network Analyses of DEGs

**Figure 4:**
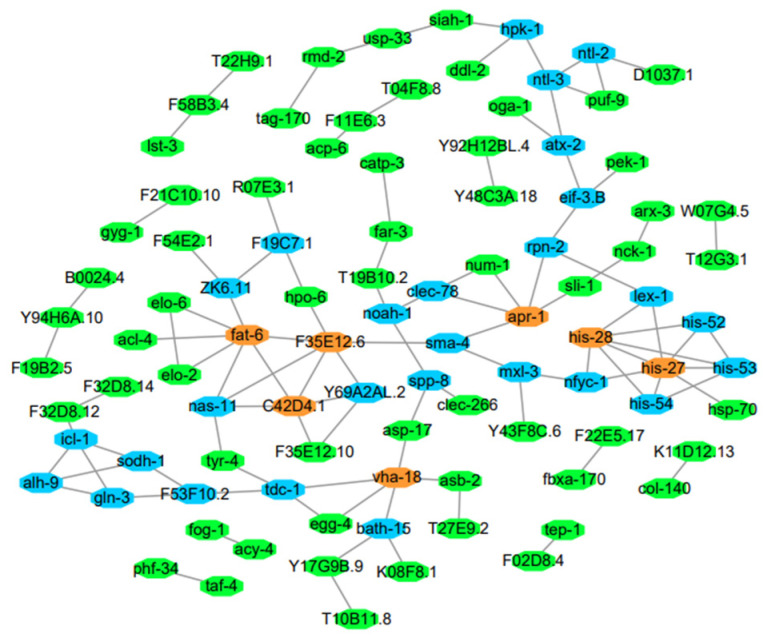
PPI networks of DEGs in *C*. *elegans* treated with astaxanthin (*p* < 0.05). Genes with degree ≥5 (each node had more than five connections/interactions) are shown in orange, degree ≥3 but <5 are shown in blue, and degree ≤3 are shown in green.

PPI networks identify genes encoding core proteins with important biological regulatory functions [22]. To further unveil the key molecular events modulated by astaxanthin, PPI was employed to analyze the interrelationship among DEGs found in astaxanthin-treated *C. elegans*. Using the STRING online database and Cytoscape software, a total of 190 DEGs (*p* < 0.05, 92 up-regulated and 98 down-regulated genes) were screened into the PPI network, resulting in 173 nodes and 105 edges. As shown in Figure 4, of the 173 nodes, 7 central node genes were identified with the filtering criteria of degree ≥ 5 (each node had more than five connections/interactions), which were *fat-6*, *F35E12.6*, *C42D4.1*, *vha-18*, *apr-1*, *his-27* and *his-28*. *Fat-6* encodes a Δ9 fatty acid desaturase, which is the rate-limiting enzyme for the biosynthesis of monounsaturated fatty acids [23]. *Vha-18* encodes the subunit H of the cytoplasmic (V1) domain of vacuolar H^+^-ATPases (V-ATPases), which are ATP-dependent proton pumps localized at the membranes of intracellular acidic organelles and plasma membranes [24] functioning in lysosomal acidification [25]. *Apr-1* is a component of Wnt signaling [26], which regulates Hox gene expression and is required for the morphogenesis of the embryo [27]. *His-27* and *his-28* encode H3 histone and H4 histone, respectively, according to Wormbase. By affecting the expression of these hub genes, astaxanthin may alter a variety of biological processes in *C. elegans*, such as fatty acid synthesis, acidic intracellular conditions, the Wnt signaling pathway and the maintenance of chromatin structure, which may contribute to the lifespan extension effects of astaxanthin.

### 3.5. Differentially Expressed miRNA in C. elegans Treated with Astaxanthin 

To identify the differentially expressed miRNA, *p* < 0.05 and |log2FC| ≥ 1 were used as cut-off criteria. As shown in Table 3, cel-miR-4814-5p, cel-miR-86-3p, cel-miR-82-5p, cel-miR-230-5p and cbn-miR-75 were significantly elevated, while cbn-miR-60 was reduced by astaxanthin treatment.

**Figure 5:**
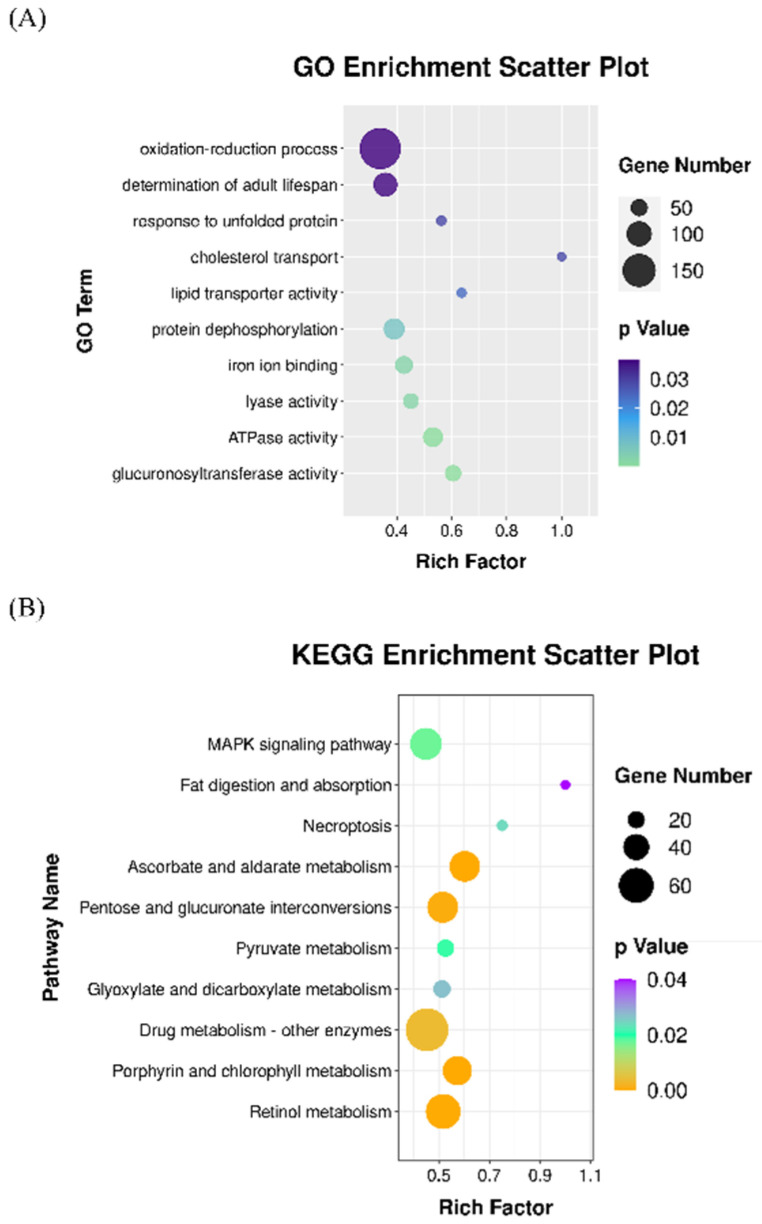
Integrant enrichment of GO terms (**A**) and KEGG pathways (**B**) of the target genes of the differentially expressed miRNA in *C. elegans* treated with astaxanthin. Rich factor is the ratio of DEG number in each group to the total number of genes identified in the GO or KEGG analyses.

The identification of potential targets is important for elucidating the regulatory network of a specific miRNA [28]. Next, the target genes of miRNAs altered by astaxanthin were predicted based on the full length of mRNA. The enrichment analyses were then performed with these target genes and a total of 368 significant GO terms and 17 KEGG pathways (Tables S5 and S6) (*p* < 0.05) were found. Among them, the determination of adult lifespan (GO:0008340), oxidation-reduction process (GO:0055114), response to unfolded protein (GO:0006986), lipid metabolism (fat digestion and absorption (ko04975), lipid transporter activity (GO:0005319), cholesterol transport (GO:0030301)), MAPK signaling pathway (ko04010) and glucuronosyltransferase activity (GO:0015020) were related to longevity and lifespan extension (Figure 5). It has to be noted that several GO terms and KEGG pathways enriched in the target genes of significantly altered miRNA, such as the oxidation-reduction process (GO:0055114), iron ion binding (GO:0005506) and glucuronosyltransferase activity (GO:0015020), were also enriched in DEGs in *C. elegans* treated with astaxanthin, suggesting that astaxanthin could affect these biological processes via the modulation of miRNA expression.

### 3.6. Effects of Astaxanthin on Lipid Metabolism in C. elegans

**Figure 6:**
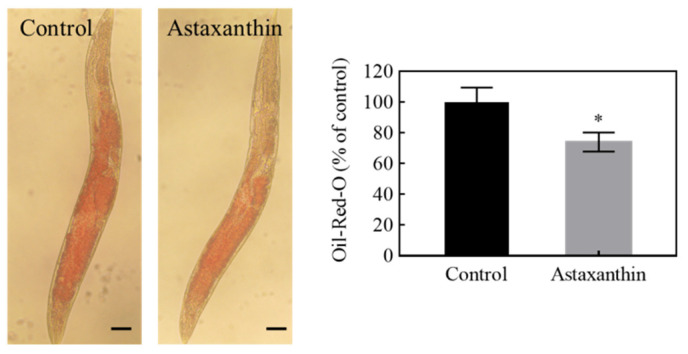
Effects of astaxanthin on fat accumulation in *C. elegans.* Worms synchronized to the adult stage were treated with or without astaxanthin for 6 days and stained with Oil Red O. Image-Pro Plus was used to quantify the intensity of Oil Red O staining. Scale bar: 100 μm. Data were expressed as mean ± SE. * Significantly different from the control worms (*p* < 0.05).

**Figure 7:**
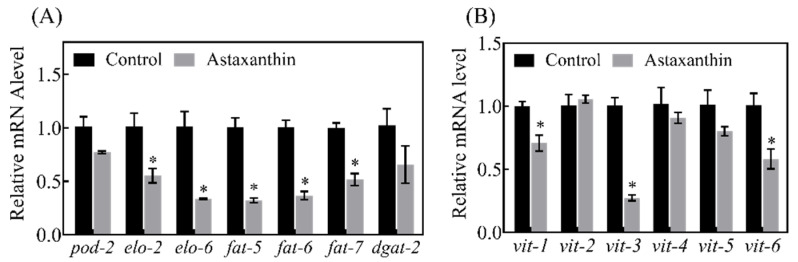
Effects of astaxanthin on the expression of genes related to lipid metabolism. Worms were treated with or without astaxanthin for 6 days. The mRNA levels of genes related to fatty acid synthesis (**A**) and vitellogenin (**B**) were determined by qRT-PCR. The expression of *pmp-3* was used as the internal control. Data were expressed as mean ± SE, n = 3. * Significantly different from the untreated worms (*p* < 0.05).

Lipid metabolism plays an important role in the aging process, the modulation of which via dietary, genetic or pharmacological interventions can affect lifespan in various model organisms [29]. The above enrichment analyses indicated that astaxanthin treatment might affect lipid biosynthesis and transport via altering the transcriptional profile of *C. elegans*. To test this prediction, the fat accumulation in *C. elegans* treated with astaxanthin was examined by Oil Red O staining. As shown in Figure 6, astaxanthin significantly reduced fat accumulation in the nematodes. The expression of genes related to fatty acid biosynthesis and lipid transport was further explored. In *C. elegans*, triglyceride synthesis relies on reactions catalyzed by a series of enzymes including acetyl Co-A carboxylase (ACC/*pod-2*), fatty acid synthase (FASN/fasn-1), fatty acid elongases (ELO/*elo*), stearoyl-CoA desaturase (SCD/*fat*) and diacylglycerol acyltransferase (DGAT/*dgat-2*) [30,31]. ACC and FASN catalyze the first and second step in the de novo fatty acid biosynthesis [32]. ELO facilitates the elongation of saturated and unsaturated fatty acids [33]. DGAT and SCD catalyze the rate-limiting step of monounsaturated fatty acid synthesis [34] and triacylglycerol synthesis [35], respectively. As shown in Figure 7A, the expression of key genes in fatty acid synthesis, *elo-2*, *elo-6*, *fat-5*, *fat-6* and *fat-7*, was significantly down-regulated in *C. elegans* treated with astaxanthin. Moreover, astaxanthin treatment significantly decreased the expression of *vit-1*, *vit-3* and *vit-6*, genes encoding vitellogenins (Figure 7B), which are lipoproteins functioning in lipid transportation from intestine to gonad for yolk formation in *C. elegans*. Yolk is widely distributed throughout the body when reproduction is ceased with age, leading to ectopic fat deposition in non-adipose tissues, which causes age-associated lipotoxicity and loss-of-tissue functions [36]. The accumulation of triglyceride is accompanied by the increase of vitellogenin levels in aging nematodes [37], while the inhibition of yolk formation by the RNAi of *vit-5/6* extends lifespan of *C. elegans* [38]. Thus, astaxanthin treatment might decrease fat accumulation and prolong lifespan in *C. elegans* by repressing the expression of genes important for fatty acid and vitellogenin synthesis.

## 4. Discussion

The anti-aging effects of astaxanthin have been attributed to its antioxidant property and the modulation of key genes in the IIS signaling and TOR pathway [6,7]; however, the exact mechanisms are still unclear. In this study, we examined the alterations of mRNA and miRNA in *C. elegans* treated with astaxanthin using RNA-seq. A total of 190 mRNAs and 6 microRNAs (miRNAs) were significantly changed by astaxanthin treatment in *C. elegans*.

As expected, the results showed that astaxanthin significantly up-regulated pathways related to longevity regulation. The enrichment analyses of GO terms and KEGG pathways demonstrated that the most prominent function of the genes altered by astaxanthin treatment was related to innate immunity, lipid metabolism, heat shock response, cytochrome P450 family and UGTs (Figure 3). It is well known that stress, immunity and aging are inter-related [39]. In fact, the most dramatic transcriptional changes that occur during aging are associated with immunity [40]. Heat-shock response, which declines in potency over the lifetime, not only has a significant relationship with longevity but also mediates pathogenic resistance against bacterial infection [41]. Genes encoding small heat shock proteins, which are transcriptionally activated by DAF-16 and HSF-1, make a substantial contribution to lifespan extension [42]. To cope with toxic stress, *C. elegans* utilize a series of enzymes for detoxification. Cytochrome P450s are the principle phase 1 enzymes for xenobiotic metabolism, catalyzing the oxidation of environmental chemicals as well as endogenous compounds, such as steroids and fatty acids, facilitating their degradation or elimination [43]. UGTs are the major effectors of phase 2 metabolism, which catalyze conjugative reactions, increasing the solubility and excretion of toxic compounds. The efficacy of the detoxification system is closely linked to the aging process and longevity [21]. Hence, astaxanthin may extend the lifespan of *C. elegans* by improving their capability to cope with environment insults and endogenous stress conditions.

As signaling molecules, lipids play crucial regulatory roles in aging and longevity. A series of genetic pathways (such as IIS) that affect lifespan are linked to lipid metabolism and lipid signaling [36]. Sphingolipids are highly conserved lipid components of the cell membrane, which are involved in the formation of lipid raft. It has been shown that sphingolipid metabolism plays a key role in regulating development and aging in *C. elegans* [44]. Thus, the alteration of sphingolipid metabolism might contribute to the anti-aging effects of astaxanthin. Among the DEGs related to lipid metabolism, *fat-6* was one of the hub genes shown in PPI network (Figure 4). It was shown by qRT-PCR that astaxanthin repressed the expression of *fat-6* along with several other key genes associated with fatty acid synthesis, such as *fat-5* and *fat-7* (Figure 7A). Consistently, the fat accumulation in the worms was significantly reduced by astaxanthin treatment (Figure 6). It has been shown that the elongation and desaturation of fatty acids are integral to various phenomena, such as ontogeny and lifespan. In *C. elegans*, *elo-2* RNAi significantly increases lifespan [45]. The *fat-5* mutant shows a slight but significant lifespan extension, while the lifespan of *fat-6* or *fat-7* mutant is not significantly different from the control [46]. Previous studies suggested that down-regulation of *elo-2* or *fat* genes inhibited fat accumulation [45,47]. Thus, reduction of *elo* and *fat* gene expression might contribute to the decrease of fat accumulation in *C. elegans* treated with astaxanthin. It is suggested that the ectopic accumulation of fat in elderly *C. elegans* causes lipotoxic effects that result in the decline of tissue function [48]. Therefore, astaxanthin treatment might be able to ameliorate the aging process in *C. elegans* by reducing fat accumulation, leading to lifespan extension. As SCDs are responsible for the generation of vital unsaturated fatty acid components of membrane, triple mutant of *SCD*/*fat* genes is lethal [46]. Among the double mutants, the *fat-6*;*fat-7* displays the most significant changes in the composition of unsaturated fatty acids and extensive physiological defects, such as slower growth, reduced movement and lower fertility [49,50]. Currently, it is unknown whether the reduced expression of *fat* genes changes the fatty acid composition and membrane function in *C. elegans* treated with astaxanthin, but no significant alterations were observed in body size and fertility (data not shown). Additional research needs to be carried out to understand how astaxanthin modulates lipogenesis and lipid homeostasis. Moreover, the expression of *vit-1*, *vit-3* and *vit-6*, which function in lipid transport, was down-regulated by astaxanthin treatment (Figure 7B). It has been demonstrated that the loss of *vit-6* promotes longevity in *C. elegans* [38]. Therefore, the down-regulation of these genes crucial for lipid synthesis and transportation was an important factor for the reduction of fat accumulation and lifespan extension in astaxanthin-treated *C. elegans*.

PPI network analyses also found *his-27* and *his-28* as hub genes, which encode histone protein (Figure 4). Histones are essential components of eukaryotic chromosomes [51]. Each nucleosome, which is basic unit of chromatin, is composed of an octamer of core histones (two each of H2A, H2B, H3 and H4) [52]. It has been demonstrated that there is a global loss of histone proteins during aging [53]. Here the results showed that the expression of *his-27* and *his-28* was decreased after astaxanthin treatment, while the expression of other histone genes, including *his-52*, *his-53* and *his-54*, were increased (Table S2). Currently, it is unknown whether the global content of histones is affected by astaxanthin treatment. The post-translational modifications (PTMs) of histones, such as acetylation, phosphorylation and methylation, play fundamental roles in transcription and many other DNA processes [54]. Both the levels and the PTMs of histones are linked to lifespan regulation and possibly influence lifespan through transcriptomic changes, resulting in the expression of key longevity genes [55]. It was found by qRT-PCR that the expression of *pcaf-1*, which encodes PCAF/GCN5-like histone acetyltransferase, was decreased by astaxanthin treatment (Data not shown). The inhibition of histone acetyltransferase GCN5 prolongs the replicative lifespan of yeast and reduces senescence in human cell lines [56]. It would be interesting to find out in the future studies whether the reduced expression of *pcaf-1* leads to lower histone acetylation and promotes longevity in astaxanthin-treated *C. elegans*.

The RNA-seq results showed that astaxanthin treatment significantly altered the levels of several miRNAs, including cel-miR-82-5p and cel-miR-230-5p (Table 3). MiR-82 belongs to miR-58 family that is by far the most abundant family in *C. elegans*, accounting more than 30% of all total miRNAs, which is composed of four highly abundant members, miR-58.1, miR-80, miR-81 and miR-82 [57]. In *C. elegans*, the level of miR-82 is significantly decreased with aging [58], while the loss of miR-82 shortens lifespan [59], suggesting that miR-82 has a role in longevity. The expression of miR-230 is decreased from Day 0 to Day 12 in *C. elegans*, suggesting that it may serve as a regulatory molecule in aging [60]. Thus, the increased expression of miR-82 and miR-230 may contribute to the longevity promotion effect of astaxanthin. Currently, the target genes of miR-82 or miR-230 in astaxanthin treated *C. elegans* were unidentified, the discovery of which will clarify how astaxanthin functions through modulating miRNAs. Enrichment analyses showed that several biological functions, such as oxidation-reduction process and glucuronosyltransferase activity, were identified in both DEGs and the predicted targets of significant miRNAs (Figure 3 and Figure 5). Therefore, it is possible that astaxanthin modulates these biological functions via modulating miRNA expression; however, further experiments are required to verify this.

In this study, the higher concentrations of astaxanthin were less effective for increasing the lifespan of *C. elegans*. Similar dose-response effects on lifespan were found for other bioactive compounds [61,62]. It is well established that both nutrients and beneficial/toxic chemicals may produce opposite consequences for an organism depending on the doses. To achieve beneficial effects, there is an optimal dose range, concentrations higher than which may be less beneficial or even lead to toxic effects. It was possible that higher concentrations of astaxanthin induced additional alterations in gene expression or biological function that counteracted the longevity promoting signals and, consequently, were less effective in lifespan extension.

## 5. Conclusions

**Figure 8:**
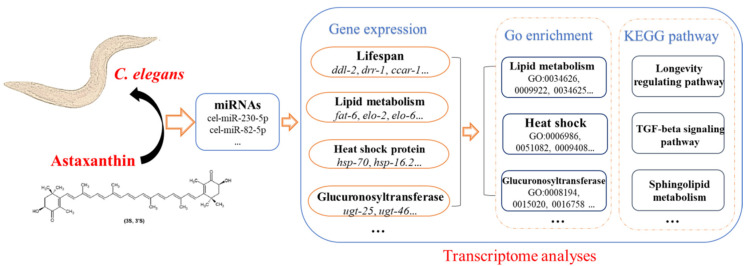
Transcriptomic analyses of *C. elegans* treated with astaxanthin.

In summary, the results of the genome-wide transcriptional profiling of N2 worms treated with astaxanthin suggested that the longevity promotion mechanisms of astaxanthin may be related to its modulation of gene expression in lipid metabolism, environmental resistance, detoxification and histone regulation. The up-regulation of miRNAs, such as cel-miR-230-5p and cel-miR-82-5p, may also be involved in the lifespan extension by astaxanthin in *C. elegans* (Figure 8). However, the specific function of individual miRNAs and their regulatory networks need further investigation to elucidate their role in astaxanthin-induced longevity.

## Figures and Tables

**Table 1:** Statistical analyses of the lifespan of *C. elegans*.

Conc. (μg/mL)	Mean Lifespan (Days)	Median Lifespan (Days)	Max. Lifespan (Days)
0	15.4 ± 0.3	15 ± 0.3	21.0 ± 1.0
5	16.3 ± 0.3 *	16 ± 0.4	22.7 ± 1.2
10	16.5 ± 0.4 *	16 ± 0.4 *	24.7 ± 0.3 *
20	17.3 ± 0.3 *	17 ± 0.5 *	23.7 ± 1.6 *
40	16.1 ± 0.3	16 ± 0.3	23.7 ± 0.3
80	15.8 ± 0.3	16 ± 0.2 *	21.0 ± 2.1

Data are expressed as mean ± SE, n = 3. * Significantly different from the untreated worms (*p* < 0.05).

**Table 2:** The DEGs in *C. elegans* treated with astaxanthin.

Gene Symbols	Description	Fold Change	*p*-Value
*rmd-2*	Regulator of microtubule dynamics	4.56	2.43 × 10^−71^
*T22H9.1*	rRNA biogenesis protein RRP36	0.28	9.77 × 10^−4^
*F47E1.2*	Solute carrier organic anion transporter family member	0.49	1.09 × 10^−3^
*lex-1*	Tat-binding homolog 7	0.46	4.66 × 10^−3^
*kvs-5*	K (potassium) voltage-sensitive channel subunit 5	0.47	0.01
*clec-78*	C-type lectin	0.33	0.01
*mxl-3*	Max-like	2.09	0.01
*phf-34*	PHD finger family	0.13	0.01
*drr-1*	Dietary restriction response	0.35	0.01
*his-28*	Histone 28	0.44	0.01
*hpo-36*	Hypersensitive to pore-forming toxin 36	0.25	0.01
*oat-1*	Organic anion transporter	0.44	0.02
*rpl-37*	60S ribosomal protein L37	0.35	0.02
*F02D8.4*	Peptidase_M14 domain-containing protein	0.47	0.02
*nlp-15*	Neuropeptide-like protein	0.44	0.02
*F28H7.8*	CRAL-TRIO domain-containing protein	0.26	0.02
*H06I04.6*	CX domain-containing protein	0.48	0.03
*cyp-14A3*	Cytochrome P450 family	0.38	0.03
*linc-60*	Long intervening non-coding RNA 60	0.38	0.03
*nhr-3*	Nuclear hormone receptor family member nhr-3	0.49	0.03
*ugt-19*	UDP-glucuronosyltransferase	0.44	0.04
*F55A3.6*	FACT complex subunit spt-16	0.46	0.05

**Table 3:** The differentially expressed miRNA in *C. elegans* treated with astaxanthin.

miRNAs	Target Sequence (5′ to 3′)	Accession Number	Fold Change	*p*-Value
cel-miR-4814-5p	TTCTCAACCAACTTTGGCCACT	OP709781	inf	0.0036
cel-miR-86-3p	CTGGGCTCAGATTCGCTTAGGC	OP709782	6.78	0.018
cbn-miR-75_L+4	GATCTTAAAGCTACCAACCGGCTTCA	OP709783	3.76	0.018
cel-miR-82-5p	CGGTTTTCTCTGTGATCTACAGA	OP709784	inf	0.043
cbn-miR-60	TATTATGCACATTTTCTAGTCCA	OP709785	0.45	0.044
cel-miR-230-5p	ACTTGGTCGGCGATTTAATATT	OP709786	2.89	0.047

The miRNAs were identified using cut-off criteria (*p* < 0.05 and |log2FC| ≥ 1).

## Data Availability

Data are contained within the article and Supplementary Material.

## Supplementary Materials

The following supporting information can be downloaded at: https://www.mdpi.com/article/10.3390/antiox11112115/s1, Figure S1: Effects of astaxanthin on the expression of genes related to lipid metabolism; Table S1: The primers used for qRT-PCR; Table S2: The significant DEGs; Table S3: The significant KEGG pathways identified by enrichment analyses; Table S4: The significant GO terms identified by enrichment analyses; Table S5: The significant GO terms of miRNA target genes; Table S6: The significant KEGG pathways of miRNA target genes.
