# Supplementary material for: Astaxanthin Induces Transcriptomic Responses Associated with Lifespan Extension in Caenorhabditis elegans
Source: Antioxidants (Basel). 2022 Oct 27;11(11):2115. doi: 10.3390/antiox11112115 (PMC9687064; doi:10.3390/antiox11112115)
Supplement: Supplementary file 1 [file antioxidants-11-02115-s001.zip › Figure S1.pdf]

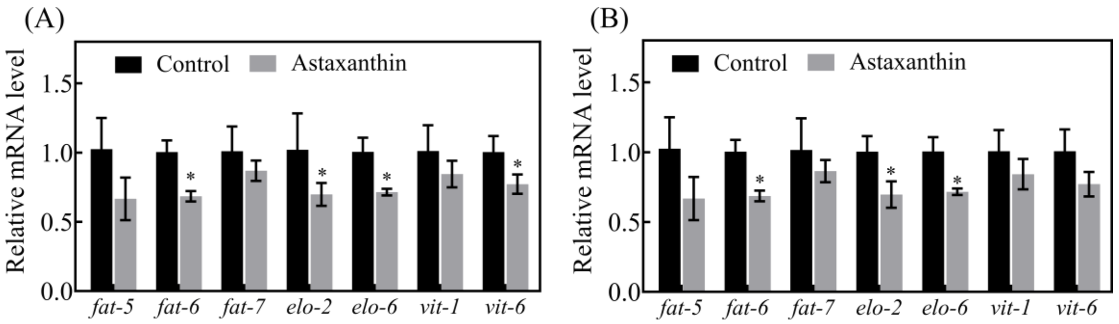

Figure S1. Effects of astaxanthin on the expression of genes related to lipid metabolism. Worms were treated with or without astaxanthin for 6 days. The mRNA levels of genes were determined by qRT-PCR and the expression of *tba-1* (A) and *cdc-42* (B) were used as the internal control. Data were expressed as mean ± SE, n=3. *Significantly different from the untreated worms (p<0.05).
